# Saccadic Adaptation to Moving Targets

**DOI:** 10.1371/journal.pone.0039708

**Published:** 2012-06-29

**Authors:** Katharina Havermann, Robert Volcic, Markus Lappe

**Affiliations:** 1 Department of Psychology, Otto Creutzfeldt Center for Cognitive and Behavioral Neuroscience, University of Muenster, Muenster, Germany; 2 Center for Neuroscience and Cognitive Systems at UniTn, Istituto Italiano di Tecnologia, Rovereto, Italy; University of Regensburg, Germany

## Abstract

Saccades are so called ballistic movements which are executed without online visual feedback. After each saccade the saccadic motor plan is modified in response to post-saccadic feedback with the mechanism of saccadic adaptation. The post-saccadic feedback is provided by the retinal position of the target after the saccade. If the target moves after the saccade, gaze may follow the moving target. In that case, the eyes are controlled by the pursuit system, a system that controls smooth eye movements. Although these two systems have in the past been considered as mostly independent, recent lines of research point towards many interactions between them. We were interested in the question if saccade amplitude adaptation is induced when the target moves smoothly after the saccade. Prior studies of saccadic adaptation have considered intra-saccadic target steps as learning signals. In the present study, the intra-saccadic target step of the McLaughlin paradigm of saccadic adaptation was replaced by target movement, and a post-saccadic pursuit of the target. We found that saccadic adaptation occurred in this situation, a further indication of an interaction of the saccadic system and the pursuit system with the aim of optimized eye movements.

## Introduction

Saccadic eye movements bring objects of interest on the high resolution fovea. They are too fast to take into account visual information online, and, therefore, have to be programmed accurately before saccade onset. An adaptation mechanism is necessary to compensate for any residual systematic deviations of the saccadic endpoint from the intended landing position. The post-saccadic visual location of the target is used to adapt saccadic amplitude. Such adaptation can be induced by an intra-saccadic target step, which induces a position error after the saccade [1]. Repetitions of this manipulation lead to an adaptation of saccadic amplitude in the direction of the target step.

Until now, only position errors have been considered as learning signals for saccadic adaptation. But, in every day life saccades occur in stable as well as in dynamic conditions. Therefore, post-saccadic errors can also arise from target movement. Moving visual targets can be tracked with smooth pursuit eye movements. Pursuit and saccades are controlled by two largely distinct brain circuits, but the focus on crosstalk between the two systems is increasing [2], [3].

In the present study, the intra-saccadic target step of the McLaughlin paradigm of saccadic adaptation was replaced by post-saccadic target movement. In this paradigm, pursuit is used to follow the target after the saccade. We can therefore expect, that the post-saccadic eye movements are under control of the pursuit system. We ask whether this situation leads to adaptation of the saccade amplitude and what learning signal for saccadic adaptation can be extracted from the post-saccadic pursuit. If the saccade system interacts with pursuit for the acquisition of the learning signal, saccades should adapt in response to the target pursuit. Otherwise, if saccadic adaptation is not supported by the pursuit system, no adaptation should occur in response to the pursued targets. We find that saccadic adaptation occurs in this situation.

Our second question regards the learning signal that drives saccadic adaptation in the post-saccadic pursuit condition. Possible candidates are the eye velocity during the pursuit, the retinal slip, i.e. target movement on the retina during the pursuit, the retinal position error of the target during imperfect pursuit, or secondary saccades. In a second experiment, adaptation to moving targets of different target velocities was used to distinguish between these signals. Adaptation was compared in conditions with slow target velocities with almost perfect pursuit to a condition with fast target velocities, in which pursuit eye velocity cannot match target velocity.

## Experiment 1

The first experiment was designed to determine whether an adjustment of saccadic amplitude occurs for post-saccadic moving targets and if this adjustment is resulting from saccadic adaptation. To do so, we test if two typical aspects of saccadic adaptation are also observable: concurrent saccadic amplitude modifications on multiple time scales and adjustments of saccade velocity and duration.

Multiple time-scales of amplitude changes demonstrate that adaptive changes are not transient within a few trials, but constitute long-term modifications in the saccadic system. Long-term effects have been previously demonstrated in saccadic adaptation [4]–[6]. After an extinction of formerly induced adaptation, readaptation is facilitated in the monkey [6]. In humans, after extensive adaptation in one direction, followed by a short phase of adaptation in the opposite direction, the former adaptation state reappeared in the testing phase [4]. Saccadic adaptation thus induces long-term learning, which is preserved over a transient period of deadaptation. Therefore, a combination of trials evoking adaptation in different directions allows the investigation of long-term adaptive changes. To demonstrate saccadic long-term adaptation also to moving targets, a block design was chosen, which combines blocks of inward and outward target movement, separated by blocks of trials in which the target simply disappears after the saccade, i.e., in which visual feedback is prevented (target-off trials). Amplitude changes can be compared on two different time scales. Short-term changes can be observed within one block, whereas long-term effects can be revealed by a comparison between blocks. Between two blocks of the same movement direction, blocks with targets moving to the opposite direction as well as target-off blocks occur. Long-term effects therefore demonstrate a memory of previous learning, which is a clear indicator for saccadic adaptation.

Secondly, saccadic adaptation induces distinct modifications of the saccade dynamics [7]. Those changes are peak velocity decrease for inward adaptation and duration increase for outward adaptation. These characteristic changes in saccadic dynamics are therefore an indicator for saccadic adaptation. Thus, we directed our focus to the analysis of changes in the saccade dynamics to show the close similarity in oculomotor learning between the adaptive changes induced by the adaptation to static post-saccadic targets and those induced by the post-saccadic moving targets.

## Materials and Methods

### Ethics Statement

Before starting the experiment participants gave their informed verbal consent in accordance with the Declaration of Helsinki and the guidelines of the local ethics committee (Department of Psychology, University of Muenster, Germany), which approved this study. The local ethics committee considered that a verbal consent was appropriate for the present behavioural study. Before his or her first experimental session, the experimenter explained the task to each subject. The statement of informed consent was noted by the experimenter. This procedure was approved by the local ethics committee.

### Subjects

12 subjects took part in the first experiment (2 males, all right handed, mean age 23 years). All of them performed the experiment twice. Successive sessions with the same subject were separated by at least 24 hours.

### Stimuli and Recording Set-up

The subject sat at 57 cm distance from a 22″ monitor (Eizo FlexScan F930). This resulted in a visual field of 40 deg×30 deg. The room was completely dark. A transparent foil reduced the luminance of the monitor by two log units and prevented the visibility of the monitor borders. On the monitor stimuli were presented with a refresh rate of 120 Hz and a resolution of 600×800 pixels. The stimuli were white squares, 0.75 deg by 0.75 deg, with a luminance of 0.5 cd/m^2^. Eye movements were recorded with the EyeLink 1000 system (SR Research, Ltd., Canada) at 1000 Hz sample rate. For all subjects the left eye was recorded. Viewing was binocular. The subject’s head was stabilized with a chin rest.

### Experimental Procedure

The subject was instructed to execute an eye movement to an appearing target and follow the target after that. The subject was on the same time informed, that the post-saccadic following might not always be successful and that he or she should do it within their natural ability. It was stressed that they should execute the saccade to the target as thoroughly as possible.

Every adaptation session consisted of five repetitions of a block design. Each repetition contained an outward and an inward adaptation block of 20 trials, interspersed with blocks of 20 target-off trials, in which no post-saccadic target appeared. The target-off blocks were deadaptation phases.

**Figure 1:**
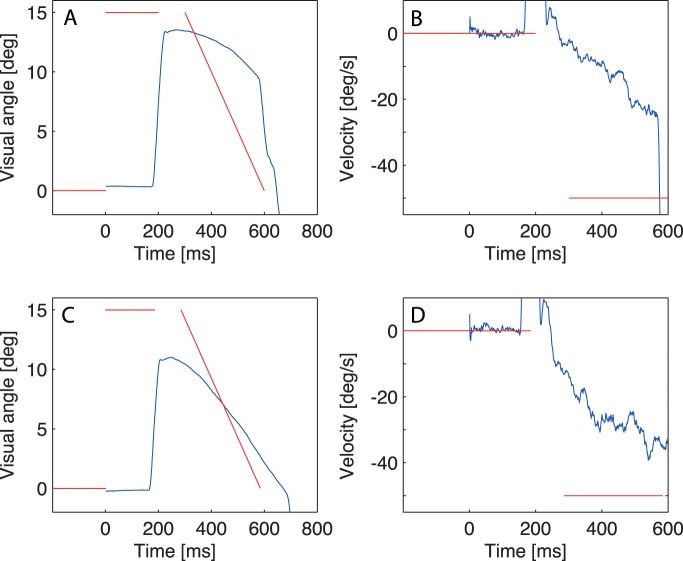
Example trials. Two example trials from an inward block. The target position and velocity are shown in red, the gaze position and velocity are depicted in blue. (A,C) Position trajectory; (B,D) Velocity. In the early trial (A, B) weak pursuit is visible, one corrective saccade occurs. In a later trial (C, D) stronger pursuit and predictive pursuit are involved.

Figure 1A shows the events during an adaptation trial. The adaptation procedure followed a modified McLaughlin scheme [1]. The trial started with a fixation point. The saccade target appeared 15 deg to the right of the fixation point after a fixation duration of 1000 ms plus a random delay of up to 300 ms. In the graph, the time is aligned to target onset. Simultaneously with the appearance of the target the fixation point was turned off. The subject was instructed to make the saccade as soon as the target appeared. When the gaze exceeded a distance threshold 2.5 deg right from the fixation point the saccade target disappeared. After 100 ms the post-saccadic target reappeared at the former target position. In the McLaughlin paradigm, a position error would be added to the target position after reappearance. Here, instead of a displaced target, a moving target was introduced. The 100 ms delay after saccade detection assured that the elicited saccade was finished by the time of the presentation of the post-saccadic target. This way, the presented post-saccadic target was perceived as a whole. The target reappeared at the former position, but started to move with a high velocity towards or away from the fixation point. The moving target induced pursuit behavior. The outward moving target disappeared after 300 ms and 15 deg at the invisible monitor borders, the inward moving target disappeared after 500 ms and 20 deg. After a further 200 ms the fixation point of the next trial appeared.

Some aspects of this paradigm resemble a pursuit step-ramp stimulus [8], [9]. Both paradigms consist of a target step, followed by a target movement. However, the two paradigms are very dissimilar with respect to the start time of the target movement and the corresponding behavior of the oculomotor system. Whereas a Rashbass stimulus combines a step and a ramp to suppress a saccade before the pursuit behavior, in our trial design, the subject first has to execute a regular saccade to the target to experience post-saccadic target motion.

Next to these adaptation trials each adaptation session contained target-off trials. In these target-off trials, as in the adaptation trials, the saccade target appeared 15 deg to the right of the fixation point after a fixation duration of 1000 ms plus a random delay of up to 300 ms. When the subject initiated the saccade and the eye position crossed the threshold right from the fixation point the saccade target disappeared. Only after 800 ms the target reappeared at the momentary eye position for 500 ms. It is known that target reappearance does not affect saccadic adaptation at such late times after saccade offset [4], [10]–[12]. After an additional delay of 700 ms the fixation point for the next trial appeared.

Furthermore balancing trials were interspersed to prevent the shortening of the latency and to avoid the occurrence of express saccades. In a balancing trial a 5 deg saccade to the left was evoked. The timing was identical to an adaptation trial, except for the target movement. The post-saccadic target just stayed stable for 500 ms. 12.5% of the trials were balancing trials. The latency was 171±49 ms.

### Data Analysis

Eye movements detected by the EyeLink software were used for analysis. These involved a 22 deg velocity threshold and a 4000 deg/s^2^ acceleration criterion. All saccade amplitudes and peak velocities are corrected for eventual predictive pursuit movements in response to the post-saccadic target motion using the method of [13]. We estimated the intersaccadic pursuit velocity by averaging pre-saccadic pursuit (55 ms to 25 ms before saccade start) and post-saccadic pursuit (25 ms to 55 ms after saccade end). The pre-saccadic pursuit component was very small, affirming the restriction of the influence of the target movement to post-saccadic times (upper quartiles: inward 0.63 deg/s; outward: 1.18 deg/s). The average intersaccadic pursuit was subtracted from the amplitudes and peak velocities. The duration was expected to be basically unmodified by pursuit. Saccades longer than 100 ms and shorter than 20 ms and smaller than 3 deg were excluded from analysis, all in all these were less than 8% of all trials.

### Statistical Analysis

The changes in saccadic amplitude and dynamics were tested short-term (within blocks) and long-term (between blocks). The first four trials in a block were averaged to measure the early-in-block amplitude, which represented the unadapted state. The last twelve trials were averaged to obtain a measure for the late-in-block amplitude, which reflected the adapted amplitude in this block. In a two factor repeated measures ANOVA the first factor was therefore the temporal occurrence within one block, which could be early-in-block or late-in-block. In this factor the short-term behavior was reflected. The block was introduced as the second factor, which showed the long-term behavior. Separate two factor ANOVAs were conducted for inward and outward adaptation blocks. The two repetitions of the subjects were averaged.

## Results

### Amplitude Modifications

We first analyze the amplitude modifications in response to the moving targets. Figure 1 shows eye position (1A, C) and eye velocity (1B, D) for two example trials of an inward block. The first example in Fig. 1A,B shows a trial early in the block, when the saccade was not yet adapted. The post-saccadic target movement induces a secondary saccade in the direction of the target movement. In a later trial of the same block the amplitude of the primary saccade was significantly shortened and predictive pursuit occurred before the onset of the post-saccadic moving target (Fig. 1C, D). The predictive pursuit allows a better match of eye position and target position (Fig. 1D). In this trial, good pursuit occurred. In the last trial of all blocks pursuit gain overall reached about 0.3 for inward movement and 0.2 for outward movement. Saccadic adaptation is reflected in the amplitude of the primary saccade, on which we focus the further analysis.

The block design of the present experiment allows the study of two time scales for adaptive changes of saccade amplitude. Whereas within one block, short-term learning develops on a time scale below 20 trials, between the blocks long-term learning at a time scale greater than 80 trials becomes apparent.

**Figure 2:**
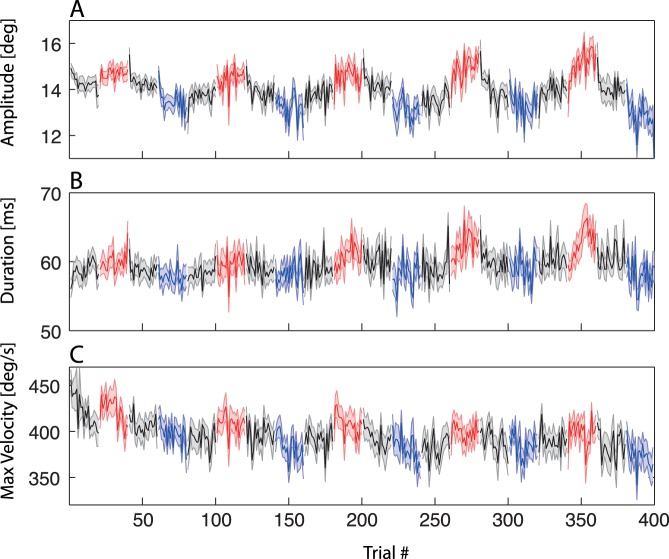
Average amplitudes, durations, and peak velocities. Averaged amplitudes (A), durations (B), and peak velocities (C) over the course of the experiment. Shaded areas show standard errors. Outward trials are marked in red, inward trials in blue. Target-off trials are shown in black.

Figure 2A shows the overall subject average of the amplitude time course. Trials with post-saccadic target outward movement are shown in red, the inward trials in blue. Target-off trials are shown in black.

It is clearly visible in this graph that short-term amplitude modification occurred. In the outward blocks, the saccade amplitude increased within one block. Accordingly, in the inward blocks the amplitude decreased within one block. Secondly, in the target-off-blocks, these amplitude modifications decayed. In the inward-target-off block the reduced amplitude increased again. In the outward-target-off block, the lengthened amplitude reached again a value, which was similar to the amplitude at the beginning of the experiment. In sum, saccadic amplitude changed in the direction of target movement.

Furthermore, long-term modifications can be observed when comparing identical blocks at different times of occurrence. Whereas the amplitude increase was small for the first outward block, it appeared stronger in later outward blocks.

To test the modifications observed within and between the different blocks, early trials and late trials of all blocks were compared in a repeated measures two factor ANOVA. This arrangement of factors describing two time scales allows to distinguish between two kinds of long-term behavior: first, a long-term main effect describing a monotonous change throughout the whole experiment, and, second, a facilitation of adaptation within later compared to earlier blocks [6]. In this latter case, relearning of, for example, inward adaptation is faster in later blocks than in earlier blocks due to some memory of the learning process. This kind of memory would not change initial gains in the different blocks. Only stronger adaptation would be expected for later blocks. In this case, a short-term main effect would occur together with an interaction of short-term and long-term behavior.

In response to the inward moving target, the amplitudes were reduced short-term (main effect: F(1,11)  = 9.8, p = 0.01) and slightly long-term (main effect: F(4,44)  = 2.49, p = 0.05). The long-term decrease in saccadic amplitude is a monotonous long-term behavior. For outward adaptation, the amplitude increased short-term (F(1,11)  = 9.6, p = 0.01). Whereas there was no overall long-term effect (main effect: F(4,44)  = 1.51, p = 0.21), an interaction was found (F(4,44)  = 3.85, p = 0.01). The early amplitudes in outward adaptation were comparable for all blocks, but for later blocks the amplitude increase was stronger. Therefore, in later blocks, adaptation was facilitated by earlier blocks like described in [6].

The target-off blocks between the outward and inward blocks constitute deadaptation phases. The ANOVA with the same factors used in the previous analyses revealed the following deadaptation effects. After outward adaptation a short-term amplitude decrease arose (F(1,11)  = 44.2, p = 0.0001) compensating for the prior saccade lengthening. For inward adaptation, only weak deadaptation was found (F(1,11)  = 3.69, p = 0.08). This mirrors the fact, that over the whole session, the amplitude was decreasing. The decrease of saccade amplitude in the inward blocks did therefore not recover completely in the deadaptation phase. Beside this, the occurring changes in saccadic amplitude decayed.

### Durations and Peak Velocities

**Figure 3:**
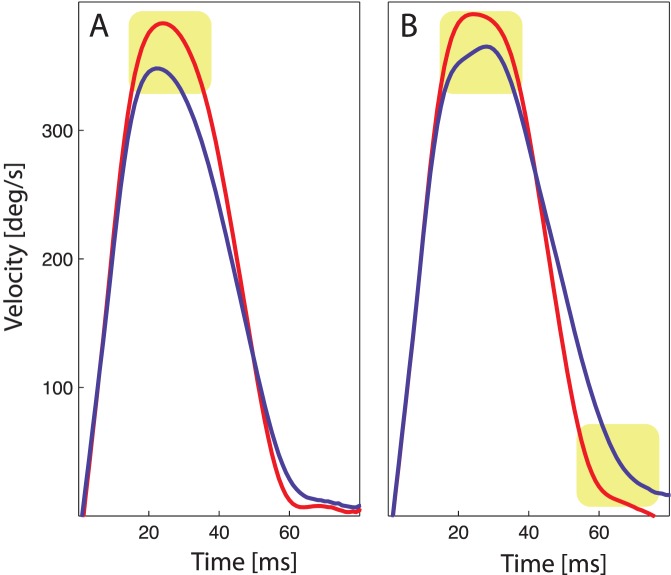
Absolute changes in velocity profiles. Absolute changes in saccade profiles for inward adaptation (A) and outward adaptation (B). The overall subjects average of saccadic eye velocity of the first four trials in red is compared to the average eye velocity of the last four trials in blue. On the x-axis the time from saccade start is depicted, on the y-axis the eye velocity. Areas of peak velocity decay and duration increase are marked with a yellow background shading.

Changes in saccadic amplitude induced by saccadic adaptation are generally accompanied by changes in saccadic dynamics. Whereas saccadic adaptation for inward adaptation mainly decreases peak velocity, outward adaptation increases duration [7], [12], [14]. This behavior was also found in our data. Figure 2B shows the development of saccade durations analog to the changes of saccadic amplitude in a group average. In Fig. 2C the changes in the peak velocities are depicted. Inward adaptation was accompanied by changes in peak velocity but no change in duration. The peak velocity decreased short-term as well as long-term (F(1,11)  = 26.0, p<0.0001, F(4,44)  = 3.3, p = 0.01). In response to the outward moving targets duration increased short-term (F(1,11)  = 27.6, p = 0.0002) and long-term (F(4,44)  = 4.9, p = 0.002). The short-term increases in duration were stronger for later blocks, paralleling the changes in amplitude (F(4,44)  = 3.7, p = 0.01). Furthermore, for outward adaptation a decrease in peak velocity was visible short-term as well as long-term (F(1,11)  = 12.2, p<0.01; F(4,44)  = 3.5, p = 0.01). This decay in peak velocities in outward adaptation demonstrates the monotonic decrease of saccade amplitude, found as a main long-term effect. A peak velocity decay was also present in the outward blocks. The absolute change of the saccadic profiles in one experiment is shown in Fig. 3. An average over the first four trials is compared to the average over the the last four trials of a given target movement direction. The decrease in peak velocity for both adaptation directions as well as the increase in duration for outward target movement is clearly visible.

We also analyzed saccade dynamics in the deadaptation blocks after inward and outward adaptation. After inward adaptation, duration stayed stable, like for the adaptation phase. After outward adaptation, the increase in duration that resulted from the prior adaptation block decayed short-term (F(1,11)  = 8.6, p = 0.01). Long-term, the duration increased over the deadaptation blocks that followed outward adaptation (F(4,44)  = 3.5, p = 0.01). This means, that the facilitated increase in saccade duration did not completely recover in the deadaptation phase. The maximum velocity in the deadaptation phase mainly mirrors the general decrease in saccadic amplitude as well. After inward adaptation, where the amplitude strongly decreased, no further changes occurred. After outward adaptation, a further decrease in the peak velocity was visible short-term and long-term (F(1,11)  = 10.6, p<0.01, F(4,44)  = 6.1, p<0.0001).

### Secondary Saccades

Since the amplitude changes observed in response to moving targets are effected by saccadic adaptation we can ask if the adaptation is induced by secondary, corrective saccades, which occur during the post-saccadic pursuit. Although for the standard target-step paradigm corrective saccades exert only a minor influence as learning signals [15], [16], they could be of increased importance in our experiment, where a target step is absent and a pursuit signal is present. An analysis of the occurrence of corrective saccades was thus of interest. Because each trial was followed by a saccade back to the fixation point corrective saccades for inward target movement were difficult to distinguish from saccades back to the position of the reappearing fixation point. Therefore we restricted our analysis to corrective saccades in the outward movement blocks, where this ambiguity is not present. All secondary saccades landing at spatial positions outward of the position of the first target were considered as corrective saccades. In about every third trial a secondary saccade was made. We then analyzed how the rate and amplitude of secondary saccades changed over the course of the experiment. The frequency of occurrence in the last block, where adaptation is strongest, was slightly, though non-significantly lower than in the first block (pre-median rate: 0.38, post-median rate: 0.25, t-test, p = 0.06). Because the rate was generally low, and does not increase over the experiment, we conclude that corrective saccades play only minor role as learning signal. The average amplitude of the corrective saccades was 4.5 deg, decreasing from the first to the last block (pre-median amplitude: 6.4 deg, post-median amplitude: 3.1 deg, t-test, p<0.001). This decrease in amplitude emphasizes their supportive role for pursuit: the amplitude of corrective saccades dropped in later trials, in which a good following of the target was already assisted by a modified amplitude of the primary saccade and a faster pursuit.

## Experiment 2

Having established that post-saccadic pursuit of a moving target leads to adaptation of saccade amplitude, we were interested in determining what post-saccadic information is used to drive this saccadic plasticity, i.e., what serves as the learning signal for the adaptive process. Candidate learning signal must come from the spatiotemporal trajectory of the gaze and the target. They include the speed (and direction) of the pursuit eye movement (a motor learning signal) or visual learning signals such as the retinal slip velocity and position errors that occur during imperfect pursuit. The quality of pursuit depends on target velocity. For low target velocities, pursuit has a high gain, i.e. the eye velocity closely matches target velocity. For high target velocities, eye velocity falls below target velocity such that over time the target moves on the retina (retinal slip) and its retinal position shifts away from the fovea (position error). Experiment 2 used different target speeds to compare amplitude changes to motor and visual candidate learning signals. As motor learning signal the average pursuit speed was calculated from the eye movement data. As visual learning signals the average position error and the average retinal slip were calculated.

## Materials and Methods

### Ethics Statement

Before starting the experiment participants gave their informed verbal consent in accordance with the Declaration of Helsinki and the guidelines of the local ethics committee (Department of Psychology, University of Muenster, Germany), which approved this study. The local ethics committee considered that a verbal consent was appropriate for the present behavioral study. Before his or her first experimental session, the experimenter explained the task to each subject. The statement of informed consent was noted by the experimenter. This procedure was approved by the local ethics committee.

### Subjects

11 subjects took part in the second experiment (3 males, one left handed, mean age 30 years).

### Stimuli and Recording Setup

Stimuli and recording setup were equal to Experiment 1.

### Experimental Procedure

Each session consisted of 20 pre-adaptation trials, 100 adaptation trials and 20 de-adaptation trials. The adaptation trials were designed analogously to the adaptation trials in experiment 1. A saccade amplitude of 20 deg was used. Three different conditions were performed, in which the post-saccadic speed of the target varied. In all conditions the target travelled a total distance of 10 degrees inward. In condition 1, the target moved with a speed of 15 deg/s for 660 ms. At this speed, high pursuit gain is expected. In conditions 2 and 3, the target moved at 30 deg/s for 330 ms, or at 50 deg/s for 200 ms, respectively. With these target velocities, pursuit velocity is expected to be lower than target velocity, giving rise to position error and retinal slip. In the pre-adaptation and de-adaptation phases, the target was stably relit and stayed for 500 ms.

Also in this experiments 12.5% balancing trials were interspersed. In a balancing trial a 10 deg upward saccade was evoked. The latency in this experiment was 171±61 ms.

### Data Analysis

Saccades smaller than 5 degrees, with latencies shorter than 10 ms or longer than 400 ms were excluded, all in all these were less than 7% of all trials. The candidate learning signals, i.e., average pursuit velocity, average position error and average retinal slip, were calculated for each trial over the duration of target presentation and averaged over each session. To calculate them, the gaze trajectory was smoothed with a 5 ms running median average, differentiated, and smoothed again with a 10 ms running median. From this data the position errors were calculated. Afterwards all saccades, as they were detected by the Eyelink criteria, were removed from the pursuit to calculate the average pursuit speed and retinal slip in each trial.

### Statistical Analysis

The amplitude change was calculated from the difference between the average pre-adaptation amplitude and the average amplitude in the last 40 adaptation trials. Each condition was first tested for significant adaptation with a t-test. In a second step, the adaptation of the three conditions was compared in a one-factor repeated measures ANOVA. The size of the learning signals was analyzed analogously. The pursuit movement and the visual signals (average position error and the average retinal slip) were considered as candidate signals.

## Results

**Figure 4:**
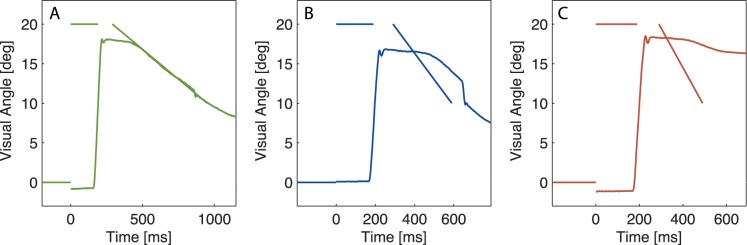
Schematic view on adaptation trials of all three conditions of experiment 2. The post-saccadic target moves with speeds of 15 deg/s (A), 30 deg/s (B), or 50 deg/s (C). On the x-axis the time is depicted in ms from the appearance of the pre-saccadic target. On the y-axis the visual angle is shown.

The second experiment considered three candidate learning signals of the adaptation induced by moving targets: eye velocity, position error, and retinal slip. In three different conditions the post-saccadic target moves inward constantly for a distance of 10 deg. The three conditions vary by their target speeds of 15 deg/s, 30 deg/s and 50 deg/s and their respective target presentation durations of 660 ms, 330 ms, and 200 ms. A schematic view of the experiment is shown in Fig. 4.

**Figure 5:**
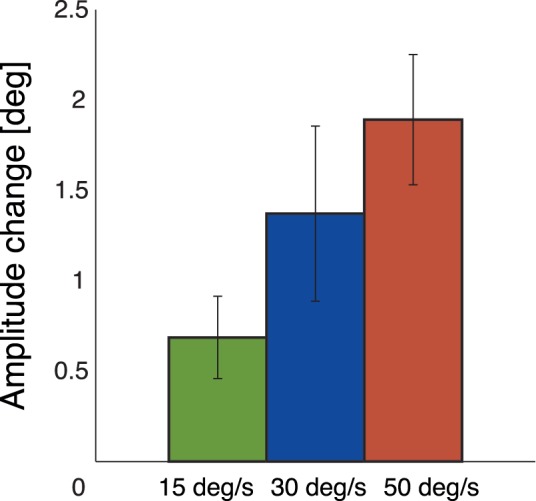
Overall subject averages of amplitude changes in experiment 2. Amplitude changes in response to targets moving at 15 deg/s, 30 deg/s, and 50 deg/s. The adaptation increased with the target speed.

We first analyze the amplitude changes in each of the three conditions. In t-tests all three conditions show significant adaptation (15 deg/s: p = 0.01, 30 deg/s: p = 0.02, 50 deg/s: p<0.001). When comparing the three amplitude changes in Fig. 5, it becomes evident that with increasing target speed the amplitude change increases. In a one-factor repeated measures ANOVA the difference between the three conditions was significant (F(2,20)  = 6.39, p<0.01). The amplitude changes are of different size, although the target distance travelled is equal in all conditions. Thus, we can conclude that the target movement itself is not the learning signal.

**Figure 6:**
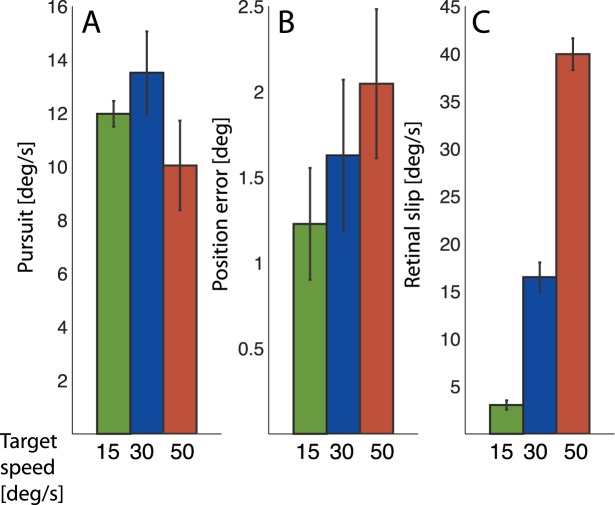
Overall subject averages of candidate learning signals. Overall subject averages of candidate learning signals of A) pursuit velocity, B) position error, and C) retinal slip. Whereas the pursuit is decreasing for the fastest target speed, the visual signals position error and retinal slip parallel the changes in saccadic amplitude.

Figure 6 plots the candidate learning signals pursuit (A), position error, (B), and retinal slip (C) for the three target speed conditions. For each trial the respective signal was calculated form the eye position data and the stimulus movement and position, and averaged over the duration of target presentation. By averaging over all trials an estimate of each candidate learning signal is calculated for each session. Figure 6A shows the eye velocity. The average eye velocity was different from zero in all three conditions (15 deg/s: p<0.0001, 30 deg/s: p<0.0001, 50 deg/s: p<0.0001). It was stronger for the 30 deg/s condition than for the 50 deg/s condition. This is contrary to the amplitude change observed in Fig. 5, where the 50 deg/s condition induced the strongest amplitude change. Therefore, the motor signal of the pursuit cannot be the learning signal for the amplitude changes. Figure 6B shows the average position error. The position error is calculated as the average difference between the unadapted saccade landing position and the position of the post-saccadic target. The reason for using the unadapted landing positions is that recent studies accumulated evidence that the position error that drives saccadic adaptation to non-moving targets is the difference between the post-saccadic retinal error and the expected error based on the hypometry of the saccade [17], [18]. In a t-test each of the conditions showed a position error different from zero (15 deg/s: p<0.005, 30 deg/s: p<0.005, 50 deg/s: p<0.001). The size of the position error increased with the target speed, paralleling the changes in saccadic amplitudes, although the differences between the different conditions did not reach significance (F(2,20)  = 3, p = 0.07).

Figure 6C shows the retinal slip 6C. Retinal slip was significantly different from zero in all conditions (15 deg/s: p<0.00001, 30 deg/s: p<0.00001, 50 deg/s: p<0.00001) and increased with target speed, paralleling the changes in saccadic amplitude (F(2,20)  = 498, p<0.0001).

We can therefore conclude, that the visual signals of position error and retinal slip can be considered as the learning signals for adaptation to moving targets.

## Discussion

In the present study, saccadic adaptation was induced by post-saccadic target movement. Short-term shortening and lengthening of saccadic amplitude was found, depending on the direction of the target movement. Their characteristics matched those of saccade inward and outward adaptation. Furthermore, two kinds of long-term motor learning effects were observed, which differed between inward and outward adaptation. For inward target movement a monotonous decrease of saccadic amplitude was found. In the outward adaptation blocks a facilitation of learning occurred.

Saccades are fast, ballistic movements. The execution of the eye movement is not visually modified online. Learning signal and saccadic modification are therefore temporally dissociated, an error signal given at the time of one saccade induces changes on the next saccade. In this study we created a paradigm, where the pre-saccadic situation was controlled by the saccadic system, whereas the post-saccadic situation was controlled by the pursuit system. This was accomplished by the presentation of a pre-saccadic static target combined with a post-saccadic target movement.

Since saccadic adaptation has some specific and revealing effects on saccade dynamics, we analyzed two time-scales of saccadic adaptation and measured saccadic dynamics. In saccadic adaptation to static post-saccadic targets various differences exist between adaptive shortening and lengthening [7], [12], [14], [19]–[22]. Also in the present paradigm, differences in the adaptation behavior were found. For outward adaptation, we could show a facilitation effect, i.e. later blocks in a session showed stronger adaptation. Inward adaptation was accompanied by a general decrease in saccade amplitude. Saccade dynamics changed in line with changes observed in adaptation to static post-saccadic targets [7]. The peak velocity was reduced for inward adaptation. For outward adaptation saccadic duration increased, whereas the peak velocity decreased. We can therefore conclude that the observed changes origin from adaptation of the saccadic system.

In the second experiment, different candidate learning signals for adaptive amplitude changes were compared. In each condition the post-saccadic target travelled a distance of 10 deg with a specific target speed. Adaptation was measured in three conditions of 15 deg/s, 30 deg/s, and 50 deg/s. With increasing target speed stronger adaptation was found. The strength of the learning signals eye velocity, position error and retinal slip were compared to the adaptation behavior. The eye velocity was strongest in the medium target speed condition of 30 deg/s, it is therefore not the learning signal for the adaptation. Both visual signals, the position error and the retinal slip increased with the target speed, paralleling the changes in saccadic amplitude. We can therefore conclude, that the visual signals, position error and retinal slip, are likely learning signals for adaptation to moving targets.

Pursuit consists of two components: smooth eye movements and catch-up saccades. The speed of smooth eye movement in pursuit can also be adapted [23] and changes in the pursuit speed after saccades have been demonstrated [24], [25]. Additionally, catch-up saccades that occur in response to the sudden onset of target movement are influenced by target velocity [26]. This change in saccade amplitude induced by the pre-saccadic target motion is also reflected in changes of saccade dynamics, which were, however, different from the changes observed in saccadic adaptation [27]. Catch-up saccades during ongoing pursuit can also adapt in response to target steps [28]. Thus, when considering the adaptation of saccades in interaction with the pursuit system two main question arise. First, to which extent are the saccades in a pursuit situation, e.g. catch-up saccades comparable to normal saccades? And second, does target movement during pursuit provide a learning signal for adaptation? Whereas the first question tries to position the catch-up saccade mechanism within the interactive system of saccades and pursuit, the second question considers the learning signals for adaptation, in first instance independently of the mechanism of catch-up saccades. The similarity of catch-up saccades to normal saccades has been addressed in a transfer study of saccadic adaptation [29]. This study showed that saccadic adaptation acquired in a static situation transfers to catch-up saccades during pursuit, suggesting a final common path for both saccade types. The integration of the catch-up saccades in the pursuit system was demonstrated by [30]. The target movement compensation of catch-up saccades was impaired after lesions of the middle temporal visual areas. The second question considers the post-saccadic influence of target movement on the saccadic adaptation system. Indications of a target movement-related saccade amplitude modification were observed in a step-ramp-ramp paradigm in monkeys, however, no such adaptation to moving targets was previously reported in humans [24], [25]. Furthermore, these two studies differ from our study primarily by one aspect: the target was moving also pre-saccadically, which might have increased the influence of predictive pursuit on the saccade.

In the present study, a pursuit situation influenced saccades to pre-saccadic static targets. In response to moving targets, saccadic adaptation was observed. This adaptation is not related to any pre-saccadic target movement. We can therefore conclude, that not only a final common pathway exists for catch-up saccades and saccades, which makes them adaptable by target steps, but furthermore does this adaptation mechanism calculate learning signals from moving targets. We further conclude that the calculation of the learning signal for saccadic adaptation is influenced by the pursuit system. The retinal slip was a much better descriptor of the observed adaptation than the position error and, as such, might be the primary input for the calculation of the learning signal. The use of retinal slip as error signal may imply an inclusion of the areas MT and MST in the saccadic adaptation mechanism.

To summarize, saccadic adaptation is induced by moving targets. Whereas in response to static post-saccadic targets only corrective saccades can be initiated to reach the target, in this paradigm post-saccadic pursuit occurs. This adaptation is therefore qualitatively different from adaptation to post-saccadic static targets, because the pursuit system controlled the eyes during the acquisition of the error signal. Nonetheless, the oculomotor system did not only induce pursuit to follow the target, but also initiated adaptive changes in the saccadic system. Our results therefore provide further evidence for an interaction between the saccade and the pursuit system.
